# Gender, money and professional identity: medical social work and the coming of the British National Health Service

**DOI:** 10.1080/09612025.2017.1328760

**Published:** 2017-06-16

**Authors:** George Campbell Gosling

**Affiliations:** ^a^ School of Social, Historical and Political Studies, University of Wolverhampton, Wolverhampton, UK

**Keywords:** Social work, professional identity, National Health Service, hospital, money

## Abstract

The arrival of the British National Health Service (NHS) in 1948 heralded significant changes for all health workers, but the establishment of a ‘free’ health service was especially meaningful for the hospital almoners—or medical social workers, as they were starting to be known—who had previously been responsible for the assessment and collection of patient payments. It was on this basis they had gained a foothold in the hospital, capitalising on gendered assumptions of financial understanding and behaviour. Yet what might have caused an identity crisis was embraced. This was a dual strategy of both repositioning the profession in alignment with the planned NHS and of asserting an enhanced professional status by distancing themselves from the handling of payment. It was an episode in the history of this distinctly female profession that speaks to women’s historic relationship with money.

The advent of the National Health Service presents both a challenge and an opportunity to almoners. For over a quarter of a century duties connected with assessment [for patient payments] and the attendant administration involved have hindered them from realizing to the full their primary function. The introduction of the National Health Service, which is generally welcomed by almoners, will free them from these burdens and enable them to devote their time to the welfare of the patient.^1^

So opened the editorial of *The Almoner: A Journal of Medical-Social Work* just weeks before the Appointed Day of 5 July 1948 when the National Health Service (NHS) came into being. All health professionals had to find their place in the new system, and the almoners were no exception. Half a century after the appointment of the first Lady Almoner, the Institute of Almoners had over 1000 active members, 717 of whom were in permanent posts in British hospitals.^2^ Most were working in the old voluntary hospitals, but the war had increased the number working in public hospitals too, often opting for the title of *medical social worker* over *almoner*, implying a greater welfare focus in their work. This emergent healthcare profession—which granted women an unusually high-status position within the male-dominated medical world—was facing an uncertain time, searching for a role within the new health service. Across a series of professional publications, a clear message was put across: that the introduction of the NHS, free at the point of use, would liberate them from an administrative burden that distracted them from their real work. It was voiced by the profession’s leading figures and their allies, from sympathetic hospital administrators to the Royal College of General Practitioners. Even Aneurin Bevan himself, the NHS’s founding Health Minister, paid lip service to the idea. Just as he said the NHS would set the people free from financial anxiety at times of sickness, the new service was envisaged as freeing the almoners from matters of money in their working lives.

In keeping with this professional positioning, the financial side of the almoners’ work has been pushed to the margins in the writing of their history. The reality was inevitably more complex. The relationship between the financial and social work sides of the profession was far more than simple distraction. In fact, common understandings of female expertise and authority in the field of finance in the early-to-mid twentieth century served to underpin the professional legitimacy of the almoner. While financial expertise was important in establishing the profession, the disentanglement of financial and social work thereafter was also important. This was more than an opportunistic strategy for repositioning the profession in line with the new health service, within which they hoped to secure their standing. The process of distancing themselves from the handling of money was one by which they were able to assert a higher status for the profession, following the earlier example of the medical profession.

It may have served this dual pragmatic purpose to write-off the financial side of their professional role as an unnecessary distraction from ‘genuine medical social work’,^3^ but to really understand this emergent female profession we need to recognise the important place dealing with and then distancing themselves from questions of payment had in shaping and cementing their professional identity and authority. This article will do so by critically re-examining the professional writings and debates of the 1940s and 1950s, principally by placing them within the wider context of the almoner profession’s history and position in the early to mid-twentieth-century British hospital.

## Locating the almoner

Rethinking the financial side of the almoner’s role can shed new light on three different lines of historical inquiry. The first of these broader histories within which we can locate the almoner is that of the NHS, which is currently being expanded in a variety of new directions.^4^ One of these is what might be termed ‘cultural history’ investigation in the realm of representation and meaning.^5^ The myriad meanings of the NHS being a ‘free’ health service must be central to any such intellectual endeavour. Here the responses of the almoner profession to the arrival of the NHS offer an enlightening perspective on the meaning of ‘free’ from the viewpoint of those who had previously acted as intermediaries between hospital doctor and patient, setting the price and handling the money. By examining this moment of uncertainty and opportunity within a larger professional project, we are able to interrogate the process of demonetising healthcare at the heart of the foundation of the British welfare state.

Second, questions of handling money also offer a window into the negotiation of the professional identity of the almoner, a female profession largely overlooked in the substantial attention historians have paid in recent decades to Britain’s pre-NHS hospitals. This has seen the old impression of the interwar years as simply a prelude to the NHS replaced with an appreciation of it as a distinct period in its own right.^6^ Specialisation and co-ordination across the mixed economy of healthcare have been recast in a more positive light than the previous image of pre-NHS voluntary hospitals as parochial, conservative and cash-strapped institutions.^7^ New forms of community fundraising have offered a caveat of philanthropic continuity in keeping with a wider narrative of progressive change.^8^ Meanwhile, the dual expansion of mutualistic hospital contributory schemes and public provision underpinned an increasingly universalist hospital service.^9^ Yet the Lady Almoner has not yet received her rightful place as a significant figure in the medical landscape of the early-to-mid twentieth century. What this has left under-examined until recently are the direct payment schemes that became the norm following the First World War.^10^

It is usually assumed that, once payment entered the equation, the old voluntary hospitals became essentially private hospitals.^11^ Yet commercial arrangements with the hospital making a profit were only in place for middle-class patients, who were barred from the *public wards* on the grounds that their admission would be an abuse of charity. The vast majority of patients had incomes below the *middle-class* threshold and received heavily subsidised treatment, to which they either contributed financially through membership of a mutual scheme or else they would be assessed by an almoner who would decide how much they should be asked to pay. She might ask for as much as a guinea a week, a far bigger burden than the two, three or four pence membership fee for a contributory scheme, but in many cases the almoner would significantly reduce the amount or even regularly pass patients entirely free. In the 1930s, when local authorities began taking over old poor law infirmaries as public general hospitals *en masse*, the introduction of patient payment schemes and the appointment of almoners to run them were amongst the ways they mimicked the less stigmatised voluntary hospitals. Thus, it was the almoner who determined the price tag of hospital care across the mixed economy in Britain before the NHS.^12^

Yet what history has been written by social workers has focused on the profession’s origins in Victorian London and its central administration, ignoring the influential role as gatekeeper and price setter that developed in hospitals across the country.^13^ Where historians have turned their attention to the almoner, they have opened up new avenues of inquiry. Lynsey Cullen has fleshed out our understanding of the first almoner in 1890s London, and Keir Waddington the concerns over the abuse of charity that led to her appointment.^14^ Martin Gorsky and Barry Doyle have noted the contribution of the almoner to integrating the pre-NHS patchwork of medical services, while Jane Lewis characterised the almoner as being ‘responsible for forging the crucial links between individual, family and community, and hence to the wider society and state’.^15^ Elaine Thomson has more critically highlighted the moral surveillance role of the almoner at the interwar Edinburgh Hospital for Women and Children.^16^ Bringing a rare focus to the post-war years, Chris Nottingham and Rona Dougall have examined the professional insecurities of medical social workers in the Scottish NHS.^17^ Despite the fact the very title is an allusion to the distributing of alms, however, it is only recently that the significance of the financial side of the almoner’s role for the hospital and the patient has been addressed.^18^ In this article we turn to its significance in relation to the professional identity of the almoner.

In doing so, the case of the almoner can shed some light on our third line of inquiry: the wider history of women and specifically the social meanings of their public and private relationships with money in modern Britain. A growing body of work has shown that, despite a host of legal, social and cultural obstacles, women in modern Britain and beyond have frequently been involved in the development of the stock market and other forms of financial management, commanding knowledge and gaining valuable experience of money matters in the process.^19^ These works have rediscovered the place of women within the world of business and as investors, and done much to challenge assumptions that women were less successful in these endeavours than their more numerous male counterparts.^20^ What has persisted, however, is an idea that women’s financial activities have been notably conservative and risk-averse.^21^ This was certainly an influential notion in the years following the First World War, when patriotic participation in the War Loans scheme encouraged a general advance of women as investors. These women were widely considered to treat savings and investments differently to men, with financial journalist Hartley Withers noting in 1930 ‘that wide-eyed sceptical curiosity that makes women so formidable’.^22^

Where Amanda Vickery found that being prudent and economical were advantages for women in the eighteenth-century middle-class marriage market, the case of the hospital almoner suggests the feminine expertise of domestic finances had professional purchase in the early twentieth.^23^ When patient payment schemes were introduced it was female social workers who were employed to manage them. Yet the question of why it was them rather than male financial clerks, or even the male inquiry officers they often replaced, has gone unasked. The answer speaks both to unspoken assumptions about the hospital—that it should operate as a charity rather than a business—and to those about the nature of women’s abilities, authority and expertise. A risk-averse feminine approach to professional financial matters cannot be entirely separated from frugality, thrift and resourcefulness in domestic management borne of a relationship with money as ‘a family, as distinct from an individual, resource’.^24^ It is this which sits behind the thinking that women were not only *equally* but *specifically suited* to handling patient payments.

This case study therefore suggests a separate sphere of financial expertise with public, professional cache. It nuances rather than challenges the governing assumptions of distinctly female financial behaviours. Where women were typically assumed (and still are by some) to be conservative and risk-averse, almoners capitalised on the corollary that they would therefore be cognisant of social conditions above hard-nosed financial calculations. They were not so much fighting gendered assumptions as using them to their professional advantage, and the ways in which they could do so changed in the 1940s.

## Positioning the profession in the 1940s

In 1946 Dorothy Manchée, almoner at St Mary’s Hospital in London, published her pseudo-autobiographical novel *Whatever Does An Almoner Do?* She wrote it, in part, in the hope of ‘combating the unfortunate and incorrect impression of many lay people that “she interviews patients’ relatives about fees”!’^25^ In one passage, where the almoner is talking to a young woman considering the profession, the author takes the opportunity to dispel some common misconceptions:

‘Being an Almoner,’ Ann Clavering explained, ‘is rather like being a Universal Aunt. Everyone in the hospital and many outside come with their troubles and problems for us to help solve. We find homes for babies and jobs for cripples; extra food for the hungry and extra money for the needy; glasses for those who can’t see and wheel chairs for those who can’t walk. Sometimes we are asked for paper carriers and drawing pins! The man-in-the-street usually thinks we collect money for the hospital, and that’s about the only job we don’t do!’

‘I thought you asked people what they earned and told them what to pay for their treatment,’ said Yvonne in astonishment.

‘The Almoners in some hospitals do so because they believe that they know the patients best and so can be more fair to them, but at this hospital we don’t even do that. We’re just here to help people get the best advantage from their hospital visits.’^26^

This was one of a number of writings—books, reports and speeches—on the eve of the NHS which have collectively shaped the place the Lady Almoner occupies in the history of British healthcare. Manchée used fiction to explain something also noted by the House Governor and Secretary of the Leeds General Infirmary. This was that the acceptance of duties associated with patient payment schemes, while ‘never regarded as fulfilling the wider functions for which they were trained’, had brought almoners into the hospitals and ‘given them the opportunity of proving to medical staffs, as well as to Management Committees and hospital administrators, the value of medico-social work’.^27^ The impression, typically echoed by historians, was of modern social workers cocooned within the Victorian moral interventionism of the Charity Organisation Society. Yet these writings must be understood as a product of their time. And that was a time of uncertainty, as the exact form the new health service would take—and the role that would be assigned to the various workers employed within it—remained unclear even after it was up and running. As was noted in the first editorial of *The Almoner* after the Appointed Day: ‘Almoners everywhere seem to be walking warily to avoid snares and traps into which they or their patients might fall through not knowing the contents of one of the circulars which arrive daily.’^28^

Consequently, it was important for imaginings of this still-emergent health profession and its future to be brought into line with the priorities and principles on which the new health service was being built. While the NHS Bill was being debated in parliament, a Tyneside almoner wrote urging the Institute of Almoners to speak up in the press for the special knowledge and understanding the almoner could provide to the community: ‘If a protest is not made, it seems to me possible that the almoner may come to be regarded merely as an assessment officer & as such relegated to work of little importance or even abolished.’ She may have been reassured that the ‘research & hospital work’ role of the almoner would not be ‘assigned either to the medical profession or to health visitors’^29^ when Bevan addressed their AGM two years later, saying:

The work of the doctor must be reinforced by the work of the Almoner, for it is now recognized that it is not possible for even the most skilled medical service to have its best beneficial effects upon the patient if he is harassed by domestic anxieties and by fears of the future that intelligent activity can remove. Therefore the Almoner has become a very important part indeed of the modern healing work.^30^

What exactly that role would look like in the new service, however, was still uncertain. That same year, Sir Wilson Jameson, Chief Medical Officer at the Ministry of Health, wrote that it was a time for almoners ‘to consider the fresh approach to their work demanded by the reorganization of the hospital system and made possible by their freedom from the task of financial assessment’.^31^

This was the context for the Hospital Almoners’ Association (HAA) commissioning Flora Beck, a social worker and researcher attached to the Nuffield Department of Medicine, to write *A Brief Account of Medical Social Service in Britain*. Buried deep in its ‘brief’ but authoritative pages was her explanation of the financial side of the almoner’s role, which she said had been made redundant by changes in the hospitals over the previous half century:

Since almoners first started their work the function of the hospitals has itself changed; what used primarily to be charities dispensing free medical treatment to those who could not afford a general practitioner’s fee have gradually been transformed into centres for specialist treatment which could not be obtained outside. The relationship between patient and hospital has changed accordingly, and in recent years the majority of patients have been anxious to make sure that the hospital received payment from a contributory scheme or some other source, or else themselves to pay on a business-like footing for services received.^32^

The focus of her book, however, was on forcefully presenting an entirely separate vision of the almoner as simply ‘a social worker in a medical setting’.^33^ This echoed the lengthy response issued by the HAA published in April 1945 to the Churchill coalition government’s second NHS White Paper, which explained the almoner’s contribution as helping:

the individual to understand and to use the facilities which are available for him in health and sickness, and which ensure that when he is ill he may be considered as a person with a social environment and problems all his own.

Understanding the patient’s domestic and occupational circumstances and his ‘mental make-up’, as well as ‘the varied resources of the community’, were not reasons given for the almoner’s suitability for sensitively handling payment schemes (as they had been in the past), but rather for making a distinctive contribution to the service to be provided free at the point of delivery by the new health service.^34^

Both Beck and the HAA described the almoner as working with ‘people’ and ‘patients’ rather than ‘the poor’ or ‘the group usually referred to (with increasing inaccuracy) as the “hospital class”’.^35^ While this distinction allowed for a positioning of the profession in line with the wider move towards universalism in the welfare state, not all aspects of this positioning were so prescient, reflecting the fact that not all the great promises of the mid-1940s came to fruition. The HAA called for a ‘Regional Almoner’ with a consultation and planning role at the centre of an envisaged yet ultimately unrealised integration of medical and social services.^36^ Central to this were the health centres which never materialised, commonly thought to be an ‘essential’ feature of the new service according to a 1944 questionnaire of almoners: ‘Unless health centres are rapidly developed general practitioners will not be able to take full advantage of the preventive and social services existing for the patient’s welfare’, respondents warned.^37^

While health centres posed an opportunity to use the NHS to expand the sites of their work, almoners already worked closely with convalescent homes, arranging the admission and transfer of patients, and were keen to bring them with them into the new health service. One aspect of the Churchill Coalition’s first and more radical NHS White Paper in 1944 of which almoners were especially approving was its making ‘hospital and convalescent home facilities available to all without charge at the time of sickness’.^38^ In this they could see the NHS as an opportunity to embed their best practice, and respondents described the continuation of the voluntary hospital system and the inclusion of convalescent homes in regional planning, without patients limited to homes in their own region, as crucial.^39^ Yet convalescent homes were also to remain outside the new service.

With varying degrees of prescience, therefore, the professional literature and debate of the 1940s sought to align the almoner with the imminent new health service. Given that the end of mass patient payments was at the very heart of the vision beginning to emerge even before Labour took office, the platform for this realignment was the abandonment of any element of the almoner’s professional identity relating to the financial side of their work. Losing the side of their role for which most hospitals had decided to appoint almoners in the first place could have been a crushing blow to the profession. Yet loss was presented as liberation, a reduced role as purification, allowing for a reorientation to the true purpose of the almoner—which, fortuitously, was perfectly in line with the new health service approaching fast on the horizon.

Yet it would be wrong to see financial expertise as entirely uncoupled from how almoners in the 1940s saw their own professional role within the planned NHS. In terms of patients paying for appliances, the 1944 HAA questionnaire found that: ‘Almoners appear to think that appliances of a reasonable standard of quality should be supplied free, patients requiring luxury types to pay the difference in cost.’ This prompted questions over whether there should be a charge for all repairs and replacements or only those deemed ‘unreasonable’, and whether ‘the almoner or the social security office of the future’ should assess what was reasonable and the ability of the patient to pay.^40^ The favoured solution was that ‘the almoner, in consultation with the medical officer and the instrument maker should be responsible for deciding whether repairs and replacements are reasonable’ and that ‘the almoner should be responsible for assessing the patients ability to pay any charges [*sic*]’.^41^ The almoner at the Sunderland Royal Infirmary wrote to the Institute of Almoners in 1946, questioning their stance in favour of Public Assistance Officers rather than almoners arranging any payments for surgical appliances:

Personally I feel it would be a very great pity if the appropriate officer were not someone of the standing and experience of a social worker, as I feel there are so many points with regard to provision, repair and general care of appliances which are connected with home conditions and medical conditions, and I do not feel that it is by any means purely a matter of finance.^42^

As throughout the preceding decades, the social work and financial sides of the almoner’s role could not be so easily distinguished or separated.

## Social work and financial assessment

**Figure 1.**
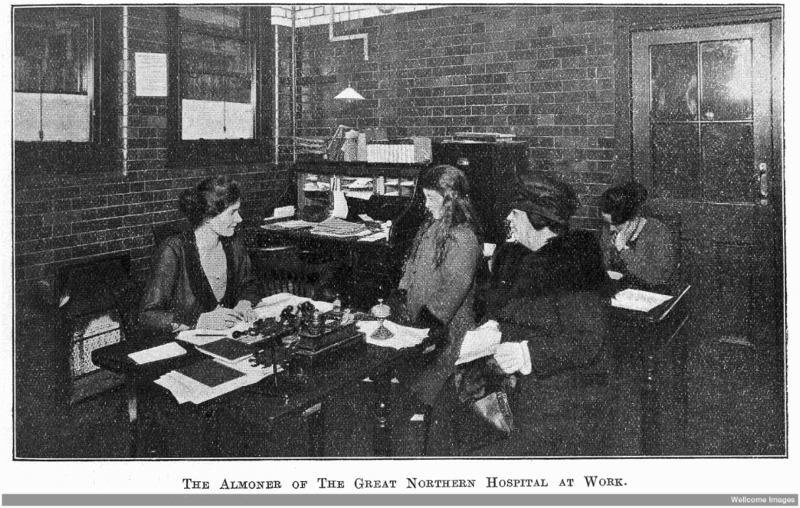
Almoner interviewing patient in London hospital, c.1920. Source: Joan Kennedy (1922) The Lady Almoner, *Hospital and Health Review*, 72, p. 133. Wellcome Images, L0015450, Wellcome Library, London.

Patients in the pre-NHS hospitals, it seems, saw the almoner first and foremost as a financial assessor. The first contact with the almoner would be an interview, taking place in her office for outpatients (see Figure 1) and usually on the ward for inpatients.^43^ One professional guide described the two tasks for the almoner in this interview:

The first is to determine whether social problems are likely to have a bearing on the patient’s illness. The second is to make the patient feel that here is a person with whom he could, if necessary, discuss his personal difficulties; someone to whom he need not mind admitting any trivial misunderstanding which had been bothering him, and to whom he could reveal serious and confidential problems without embarrassment.^44^

A third unlisted task was to use this to determine what would be an appropriate financial contribution to ask of the patient. To do this she would endeavour to establish:

the income and the chief items of expenditure of a family, the type of work on which its members are engaged, and later on such salient facts as their religion and amusements, as well as the characteristics of the home.

This could then be followed up by further investigation, where necessary and with the patient’s agreement, by seeking information ‘not only from the patient, but also through other social agencies, from relatives, from employers, or from other sources’.^45^ In the professional literature it was said to be a ‘golden rule’ that this should take place *after* the patient had seen the doctor, ensuring admission was a medical matter and such financial considerations secondary.^46^ What is less clear is whether this distinction had any meaning for the patients themselves.

While Steven Cherry has suggested that almoners ‘were often resented’, the few surviving accounts display a wider range of responses.^47^ One woman, born in 1930, has recalled:

An august but very kind lady called the Lady Almoner would come round the wards and inquire as to a patient’s financial resources. If you could you would make a contribution. If not, there was no pressure or feelings of shame. Very benign I remember.^48^

A less positive account of the almoner’s interview can be found in the memoir of Bella Aronovitch, who was moved around a variety of London hospitals in the late 1920s when suffering with appendicitis.

A few days after this first operation I had a visit from the hospital almoner. She came into the ward carrying a huge sheaf of papers and looked terrifyingly efficient. Following a few minutes’ talk with Sister she came over to me, made herself comfortable on a chair beside my bed and for the next quarter of an hour, her conversation consisted entirely of questions. She started with questions about my family. How many of us were at home? Who went to work and who were still at school? How much did I earn when I went to work? How much rent did we pay? What was our total income from all sources? etc., etc. Now all the questions were the preliminary skirmishes to the final question, which was; could my family afford to pay towards my upkeep while I was in hospital and if so, how much? … I found all those questions rather trying. However, I answered them as truthfully and to the best of my ability. As the almoner left, she told me to be sure to tell my mother to call at her office next mid-week visiting day. She then double checked with Mother on the answers to all questions.^49^

Falling somewhere between those accounts, and echoing the idea that payment was not explicitly optional, an oral history project conducted into health services in Lancashire suggests there was some understanding of the non-compulsory nature of such systems. Mrs Carson (born in 1902) recounted out-patients being treated free and inpatients being sent ‘a bill’: ‘They didn’t force you to pay it, but they would ask you to pay something or make a donation to the hospital if [you] couldn’t afford to pay the bill, which more or less everybody did something [*sic*].’ When scalded at work she ended up spending her twenty-first birthday at Lancaster Infirmary. She recalled: ‘I got a bill for it. Six bob a day.’^50^ More critically, in an article celebrating the sixtieth anniversary of the NHS, one trade unionist wrote the following:

During the second world war a woman is discharged from a south London hospital. Before she leaves the building with her young son they must see the Lady Almoner, who will determine the fees she must pay for her treatment and medicine.

The Lady Almoner sits behind a large wooden desk. She quizzes the woman about her household finances, the income and savings of everyone in the family and their daily standard of living. The interrogation over, the woman takes out her purse, pays the sum demanded, and leaves.

It is an upsetting and humiliating experience for my mother. For me, it is an early introduction to the world of means testing.^51^

There is, therefore, a significant diversity of experiences of the almoner among the few recorded. A similar range is suggested by comments in almoners’ reports that ‘[i]n almost every case the patients have been very ready to pay what they could afford’ alongside long-running complaints about the misconception of compulsory fees.^52^ There was a sense that, as Manchée observed, because the almoner was approaching the patient holistically she was most capable of making these investigations and judging the individual’s ability to pay in a ‘fair’ way.^53^ Indeed, when in December 1935 the Glasgow Royal Infirmary introduced ‘a three month experiment empowering the almoners to encourage patients who could afford it to contribute something towards their maintenance’, they believed the almoner could administer such a scheme ‘in a kindly and judicious manner without giving the slightest cause for resentment on the part of any patient’.^54^

As a profession in the process of being founded, it was crucial that its claims to authority be demonstrated in practice. The specialist skill and knowledge of the almoner was significantly not evidenced by maximising income from patient payments, but by judging the appropriate contribution to ask based on a skilful reading of the patient’s social circumstances.^55^ To this end, almoners’ reports often emphasised the numbers of patients deemed ‘unable to pay’ or even cases where payment had been offered and refused.^56^ Meanwhile, the task of collecting payments in the large working-class wards was often undertaken jointly with or delegated to clerks and administrative assistants.^57^ This likely served doubly to enhance professional standing by distancing the almoner from the actual handling of money and by putting on show their seniority over other, sometimes male, colleagues. In the unusual cases where almoners were involved in collecting payments from private patients, however, the task was rarely delegated to clerks or assistants, and never when commercial fees beyond a simple maintenance charge were being paid.^58^

The skilful administration of payment schemes and management of junior staff had strengthened the position of the almoner sufficiently that by the 1940s quite a different relationship to money could be claimed as appropriate. This echoed the objections of hospital doctors to handling payment themselves, seeing it as undermining their professional standing, with the task usually delegated to the almoners who were appointed for the purpose. Only a few decades later almoners were voicing the same objections themselves, and in doing so staking a claim to a higher professional status. This *distancing* of professionals from payment needs to be understood in three respects: professional status, purity of the medical encounter, and holding out against mission drift away from the philanthropic and welfare foundations of the hospital as a social institution.

Thus the financial and social work sides of the almoner’s role were not entirely separate, nor was one a distraction from the other. The financial was part of the domestic reality the almoner assessed and in this her expertise was not only acceptable and even useful in the hospital, but distinctly and unthreateningly womanly. ‘Educated women could win a place for themselves in the hospital world only by insisting upon their superior morality and skill’, Martha Vicinus has noted of nineteenth-century nursing reform.^59^ Meanwhile, women doctors made their case for entering the medical profession on the basis of what Vanessa Heggie calls ‘the argument from difference’, whereby the skills women could bring were inherently different from and complementary to, rather than in competition with, those of men.^60^ On which basis, for example, the medical care of women and children offered an area of medicine in which female doctors could build up their own, largely unthreatening, specialist claim to authority.^61^ In parallel, the rationale for employing female social case workers to undertake the job of assessing and collecting patient payments was the claim, usually unspoken, to three spheres of relevant expertise: knowledge of available charitable and public health and welfare services, training in the assessment of social circumstances, and understanding of domestic finance.

The situation for almoners was much the same as that for women doctors, where Heggie has rightly observed that the ‘argument about differences’ was also:

an argument about class—although these special skills were thought to be natural to women, they were not the inherent possession of *all* women. It was the educated middle-class woman who was needed to instruct, assist, treat, and supervise the inadequate mothers and wives of the poor.^62^

Indeed, this was the basis on which the almoner’s claim to professional authority rested, with such thinking pervasive amongst the governors responsible for granting almoners their professional foothold in the hospital. This was given rare voice in 1912 by the Secretary of London’s Metropolitan Hospital (where a male inquiry officer was kept on as the only male almoner in the profession’s early history) when he told a King’s Fund committee not only that ‘the Almoner’s work is ladies’ work’ but that ‘ladies are usually better if they are suited for the work. I mean ladies who are really ladies.’^63^ While the nurse could care for the sick and the midwife guide the mother through birth, the almoner could guide the family through the practical arrangements for receiving medical treatment—how to get support for the home or workplace, appliances while recovering, where to convalesce, from whom to request public or charitable assistance, and how to manage the financial implications. It was because she took responsibility for embedding the reluctantly introduced patient payment schemes into a more noble vision of care and cure that the almoner initially gained any position in the hospital.

This is not to say, of course, that the role was conceived as a purely financial one, nor that the relationship between the financial and social work sides of the role was unchanged over half a century. Indeed, four years before the first almoner was appointed, Charity Organisation Society founder Charles Loch had suggested to a House of Lords Select Committee that hospitals should appoint some form of ‘charitable assessor, or co-operator … well instructed as to all forms of relief other than medical’.^64^ The gradual acceptance of this broad welfare vision was suggested by Alan Moncrieff, Nuffield Professor of Child Health at the University of London, when he wrote in 1948 that in the half-century since the appointment of the first almoner:

medical-social work has moved away from the narrow conception of negatively preventing abuse of the hospital’s charity towards the positive aspect of contributing to the diagnosis and treatment of disease by providing the medical staff with details of the social background against which the patients’ symptoms must be judged, and his or her treatment adjusted.^65^

This was seen over a decade before the inception of the NHS, when the almoner’s department at Addenbrooke’s Hospital in Cambridge outlined their work without any mention of payment whatsoever:

Each Department of the Hospital sees the patient from a different angle. In the Almoner’s Office he is no longer the gangrenous appendix, the obstinate arthritis, or the glaucoma that has responded so well to treatment, but an ordinary human being with his background of ordinary human cares and relationships. He is for us the out-of-work trying to balance a budget that can never quite meet the household needs; an Old Age Pensioner without kith or kin; a child whose future still hangs precariously in the balance. Through the Almoner’s Office pass all the types which go to make up the Hospital world, the lonely, the misfits, the discouraged and the difficult—all through sickness or poverty, in need of some help or advice.^66^

It is certainly true that assessment for financial purposes was only one of many jobs carried out by the almoner. In some unusual cases, alternative arrangements relieved them of this responsibility altogether. For example, one former almoner at King's College Hospital recalled: ‘If you didn’t produce your [contributory scheme] voucher, or the promise of it, you put something in a box. You kind of bought a ticket and so the medical social workers there didn’t have any responsibility for assessment.’^67^ Even where they did conduct financial assessments they might not always be the highest priority. Another former almoner recalled her attitude, which she later found an easier fit in a London County Council institution than a voluntary hospital:

If I’ve got several people outside my door in out-patients with difficult problems, and others are just waiting for me to assess whether they can manage one and sixpence per attendance or not, there is no question in my mind where I’m going to spend the time.^68^

This became easier still during the Second World War, which another almoner remembered as:

a great opportunity, because the emergency medical service which was a trial run for the National Health Service, put an end to all these patients’ payments and things. The contributory schemes kept on, but they took care of so much that gradually the almoners were able to get out of that administrative chore.^69^

By reducing the focus on financial assessment, almoners were taking charge of their professional priorities and identity. This was a low-key reorientation of the profession, though it would become more assertive in the next decade.

## Positioning the profession in the 1950s

The eagerness for the almoners to abandon this aspect of their work was evident when various charges were introduced only a few years after the establishment of the National Health Service. Under Attlee’s Labour government these began with charges for dentures and spectacles, and the door was opened for Churchill’s subsequent Conservative government to bring in charges for prescriptions and hospital appliances. In 1952 the new charges for outpatients included £3 for surgical shoes, £2 10/- for a wig, and 10/- for elastic stockings.^70^ Just as the medical staff of the voluntary hospitals had been weary of directly receiving payments, for fear of sullying their hands with the dirty business of money, the almoners were adamant they would not—as some hospital administrators planned—be collecting these new charges.

The Ministry of Health told hospital management boards and committees they should ‘carefully consider what arrangements should be made for the collection of the income from these proposed charges’.^71^ Those in Windsor were amongst the many who made interim arrangements whereby:

Immediately after the Specialist has ordered the appliance etc. the patient concerned must see the Almoner, who will obtain a certificate of “promise to pay” or who will investigate or advise the patient on the exception to the general rule of payment [and the almoner’s department] shall keep all records both financial and otherwise in connection with appliances, etc., subject to charge.^72^

While this new reality was being brought about on the ground, the council of the Institute of Almoners challenged it by issuing a statement declaring ‘THAT ANY ASSESSMENT OR COLLECTION OF CHARGES UNDER THE NATIONAL HEALTH SERVICE IS NOT AN APPROPRIATE DUTY OF ALMONERS’ DEPARTMENTS AND IN NO CIRCUMSTANCES SHOULD ALMONERS (OR THEIR CLERKS) ACCEPT SUCH RESPONSIBILITIES’.^73^ One reason given, in a letter from the Secretary of the Institute to the Ministry of Health, was that there was no assessment of the patient’s circumstances, in the pre-NHS sense, to conduct.^74^ Instead, there were categories of exemption that simply needed to be confirmed: children under sixteen or attending school full-time, those receiving grants from the National Assistance Board and their dependents, and War Pensioners with an ‘accepted disability’.^75^ After months of trying not to get involved in the staffing arrangements, the Ministry of Health wrote to the Institute of Almoners stating: ‘we agree with this view’, while suggesting almoners might well ‘advise and assist’ patients making National Assistance claims. This meant, the Institute told its members, that such matters only needed to be dealt with when they arose ‘in the normal course of their work’.^76^ With the situation diffused, a note was sent to the Chief Medical Officer, Sir George Godber, telling him: ‘It seems alright now.’^77^

Refusal to return to their traditional role in assessing patients also meant they sought to have no role in ‘dealing with cases of hardship’, which they saw as the business of the new National Assistance Board. At their national association’s annual general meeting shortly after, Miss Hornsby Smith remarked:

I am sure many of you rejoice in the fact that your work is no longer associated with the extraction of money and that those other services which you render to the patient and to the National Health Service have assumed their proper place.^78^

This was not merely protestation from the social workers. A month later Ministry of Health officials were stating in no uncertain terms that, despite the new charges, there was to be ‘no requirement whatsoever for any person in the almoner’s department to assess need’ and should be no suggestion ‘that the almoner should be responsible for the collection of money’.^79^

By 1960, finance featured in the work of the almoners at Newcastle’s Victoria Royal Infirmary in around one in eight cases, but only in relation to providing or securing financial assistance. £300 was spent that year from the Almoner’s Fund ‘for needs closely connected with a patient’s hospital treatment’, fares for travelling to the hospital in the case of the larger grants. Around £70 in charitable relief from outside the hospital was arranged to help patients ineligible for National Assistance support with the cost of items including surgical shoes and spectacles. Meanwhile the almoner in the Radiotherapy Department handled over £700 of grants from the National Society for Cancer Relief, who provided weekly grants of 10/- or 15/- to an average of twenty patients at a time for additional comforts such as ‘nourishment, coal and clothing’.^80^ There continued to be a financial dimension to the work of the almoner, even if the remit had been narrowed by the NHS.

This was not, however, simply a case of adapting to the new environment. Certainly to characterise assessment of patient payments as a distraction from *pure* social work was useful in the uncertain times of the 1940s, by which time the almoner’s role could be described as social work, distinguished from other forms only in its hospital setting. Yet the premise on which it was introduced to the hospitals either side of the First World War was specifically to administer the new mass payment schemes. Where taking on the assessment of payments in the pre-NHS hospital granted the almoner a means to guarantee access for the sick poor regardless of ability to pay, even suggesting playing the same role in a universally free hospital system cast them instead in the uncomfortable light of restricting provision. In addition to facilitating this professional realignment—from mediating the traditional, class-bound philanthropic power dynamics of the voluntary hospital, to advocating for the patient in the new welfare state—shedding their financial responsibilities was also instrumental in the almoners taking charge of their own professional priorities. This came to the fore in the early 1950s when all financial duties were talked down as merely administrative, while the professional status of the almoner was asserted by this demonstration of distancing themselves from such menial monetary duties.

## Collecting payment: meanings

The economic is social, as a wealth of work by economic sociologists over the past thirty years has forcefully reminded us.^81^ It is certainly something the medical social workers of the early-to-mid twentieth century understood, and it informed their activities in assessing and collecting payments as well as advising on and securing financial support for patients whose needs were not purely medical. Money carried meaning for the patient, whether as a marker of their ability to *do their bit* and contribute to the maintenance of the hospital or to feed a family while recovering from surgery. From self-sufficiency and good citizenship to deference and dependency, almoners were navigating sensitive territories in what money meant to the patient.^82^ Yet the money they took, or declined to take, on behalf of the hospital also carried meaning for them.

For women in the hospitals of late-nineteenth- and early twentieth-century Britain, deep-seated assumptions about gendered expertise gave some degree of license and legitimacy to numerous parallel professional projects: almoners establishing a new profession within the hospital, midwives and nurses seeking to enhance their occupational standing, and those women entering the medical profession itself. In each case, claims to specifically female spheres of expertise underpinned their efforts. For the almoners, their claim was to a social understanding of finance. It was on this basis that their professional project was founded, though with one fundamental difference distinguishing it from those of the nurses or women doctors. In categorising these, sociologist Anne Witz termed the nurses’ registration campaign a strategy of dual closure, for employing both ‘usurpationary’ and ‘exclusionary’ tactics. The former in that they sought and achieved autonomy within the hospital, independent of medical men. The latter for the self-government of an occupational monopoly created over the education and entry into, as well as the infrastructure for and supply of, nursing.^83^ While these efforts targeted the enhancement of professional standing and wrestling some occupational control from male doctors and hospital administrators, they were also founded on a fuller exercise of a separate and gendered set of skills and expertise around providing care. Women doctors, by contrast, embarked on an ‘inclusionary’ strategy, ultimately achieved by parliamentary means, whereby the female professional project served as a countervailing exercise of legal power to challenge the ‘exclusionary credentialist’ restrictions to the medical education and registration of women.^84^ The gendered claims to expertise made by nurses for an entire occupation, women doctors made for specialisms within a profession.

For almoners, the task was different. Where Witz’s schema sheds light on incidents of ‘occupational closure’, the almoners were engaged in what might be better understood as an occupational opening.^85^ Their aim was not to gain entry to or enhance the standing of an established profession, but rather to found a new one. While this did involve similar challenges in asserting professional status and female claims to expertise within the male-governed institution of the hospital, the profession itself needed to be justified. By the arrival of the NHS, their aim was more akin to that of the nurses, having evolved from legitimation to enhancement. Their claim was no longer simply one of utility but was now one of professional purity. As such the professional relationship between middle-class women and money played two distinctly different roles, first in the early-century foundation of the almoner profession and later in its mid-century consolidation. Ultimately, however, both handling money and refusing to do so were deeply important for establishing, shaping and enhancing professional identity.

## Notes

1. ‘The Almoner and the National Health Service’, *The Almoner* (June 1948), Modern Records Centre, University of Warwick [hereafter MRC], MSS.378/IMSW/A/19/9/1.

2. Institute of Almoners (1948) *Annual Report*, p. 5.

3. I. F. Beck, ‘Reflections from Offley––A Summing-up of the Refresher Course. 1’, *The Almoner* (June 1948), p. 54, MRC, MSS.378/IMSW/A/19/9/1.

4. Martin Gorsky (2008) called for a range of new approaches in ‘The British National Health Service 1948–2008: a review of the historiography’, *Social History of Medicine*, 21(3), pp. 437–460, pp. 453–454. Since then the Wellcome Trust has funded a number of major projects in this area, including that led by Gorsky on ‘Health Systems in History: ideas, comparisons and policies’, which includes the NHS, and another by Dr Alex Mold on ‘Placing the Public in Public Health: public health in Britain, 1948–2010’, both at the London School of Hygiene and Tropical Medicine, and a project led by Professor Sally Sheard ‘The Governance of Health: medical, economic and managerial expertise in Britain since 1948’ at the University of Liverpool.

5. The most notable project in this area is the ‘Cultural History of the NHS’ Wellcome Trust Senior Investigator Award, jointly headed up by Professor Roberta Bivins and Professor Mathew Thomson at the University of Warwick between 2015 and 2019. For more information on the project, see http://www2.warwick.ac.uk/fac/arts/history/chm/research_teaching/research/nhshistory/ (accessed 11 September 2016). The need for more attention to such questions was emphasised by Andrew Seaton in ‘Why Researching the NHS Matters’, *History Workshop Online*, http://www.historyworkshop.org.uk/why-researching-the-nhs-matters (accessed 11 September 2016).

6. For the traditional view see Charles Webster (1988) *The Health Services since the War, Volume 1: problems of health care, the National Health Service before 1957* (London: HMSO).

7. See, for example, Nick Hayes (2012) ‘Our Hospitals’? Voluntary provision, community and civic consciousness in Nottingham before the NHS, *Midland History*, 37(1), pp. 84–105; Barry Doyle (2014) *The Politics of Hospital Provision in Early 20th-Century Britain*, Studies for the Society for the Social History of Medicine (London: Pickering & Chatto).

8. Nick Hayes & Barry M. Doyle (2013) Eggs, Rags and Whist Drives: popular munificence and the development of provincial medical voluntarism between the wars, *Historical Research*, 86(234), pp. 712–40.

9. Amongst the substantial literature on hospital contributory schemes is Martin Gorsky, John Mohan & Tim Willis (2006) *Mutualism and Health Care: British hospital contributory schemes in the twentieth century* (Manchester: Manchester University Press); Steven Cherry (1992) Beyond National Health Insurance: the voluntary hospitals and hospital contributory schemes: a regional study, *Social History of Medicine*, 5(3), pp. 455–482; Steven Thompson (2003) A Proletarian Public Sphere: working-class provision of medical services and care in South Wales, c.1900–1948, in Anne Borsay (Eds) *Medicine in Wales c.1800–2000: public service or private commodity?* (Cardiff: University of Wales Press), pp. 86–107; Barry Doyle (2010) Labour and Hospitals in Three Yorkshire Towns: Middlesbrough, Leeds, Sheffield, 1919–1938, *Social History of Medicine*, 23(2), pp. 374–92; and George Campbell Gosling (2010) ‘Open the Other Eye’: payment, civic duty and hospital contributory schemes in Bristol, c.1927–1948, *Medical History*, 54(4), pp. 475–494.

10. Some corrective to the absence of the Lady Almoner was offered by Doyle, *The Politics of Hospital Provision*; George Campbell Gosling (2017) *Payment and Philanthropy in British Healthcare 1918–48* (Manchester: Manchester University Press).

11. John Pickstone (1985) *Medicine and Industrial Society: a history of hospital development in Manchester and its region* (Manchester: Manchester University Press), p. 252; Rodney Lowe (2004) *The Welfare State in Britain since 1945*, 3rd edn (London: Palgrave Macmillan).

12. For a more detailed examination of the pre-NHS hospital payment schemes, see Gosling, *Payment and Philanthropy*.

13. Angela Simmons (2005) *A Profession and Its Roots: the lady almoners* (Michelangelo Press), https://www.kcl.ac.uk/sspp/policy-institute/scwru/swhn/publications/Simmons-A-Profession-and-ItsRoots-The-Lady-Almoners.pdf. Phyllis Willmott (1985) 1895–1945: the first 50 years, in Joan Baraclough, Grace Dedman, Hazel Osborn & Phyllis Willmott (Eds) *100 Years of Health Related Social Work 1895–1995: then–*–*now–*–*onwards* (Birmingham: BASW), pp. 1–11.

14. Lynsey Cullen (2013) The First Lady Almoner: the appointment, position, and findings of Miss Mary Stewart at the Royal Free Hospital, 1895–99, *Journal of the History of Medicine and Allied Sciences*, 68(4), pp. 551–582; Keir Waddington (1998) Unsuitable Cases: the debate over outpatient admissions, the medical profession and late-Victorian London hospitals, *Medical History*, 42(1), pp. 42–45.

15. Martin Gorsky (2001) ‘For the Treatment of Sick Persons of All Classes’: the transformation of Bristol's Hospital services, 1918–1939, in Peter Wardley (Ed.) *Bristol Historical Resource*, CD-ROM (Bristol: University of the West of England), ch. 5.1; Doyle, *The Politics of Hospital Provision*; Jane Lewis (1991) *Women and Social Action in Victorian and Edwardian England* (Stanford: Stanford University Press), p. 305.

16. Elaine Thomson (2002) Between Separate Spheres: medical women, moral hygiene and the Edinburgh Hospital for Women and Children, in Steve Sturdy (Ed.) *Medicine, Health and the Public Sphere in Britain 1600–2000* (London: Routledge), pp. 107–122.

17. Chris Nottingham & Rona Dougall (2007) A Close and Practical Association with the Medical Profession: Scottish medical social workers and the social medicine, 1940–1975, *Medical History*, 51(3), pp. 309–36.

18. Gosling, *Payment and Philanthropy*. See also Doyle, *The Politics of Hospital Provision*.

19. For good overviews and introductions, see Anne Laurence, Josephine Maltby & Janette Rutterford (Eds) (2009) *Women and Their Money 1700–1950: essays on women and finance* (London & New York: Routledge) and David R. Green et al. (Eds) (2001), *Men, Women, and Money: perspectives on gender, wealth, and investment, 1850–1930* (Oxford: Oxford University Press).

20. Alison Kay (2009) *The Foundations of Female Entrepreneurship: enterprise, home and household in London, c.1800–1870* (London: Routledge).

21. See Janette Rutterford & Josephine Maltby (2007) ‘The Nesting Instinct’: women investors and risk in England, 1700–1930, *Accounting History*, 12(3), pp. 305–327. See also David R. Green et al. (2011) Men, Women, and Money: an introduction, in Green et al. (Eds) *Men, Women, and Money*, pp. 11, 28–29.

22. Hartley Withers (1940) *The Quicksands of the City and a Way through for Investors* (London: P.S. King & Sons), p. 28 cited in Anne Laurence, Josephine Maltby & Janette Rutterford (2009) Introduction, in *Women and Their Money 1700–1950*, p. 17.

23. Amanda Vickery (1998) *The Gentleman's Daughter: women's lives in Georgian England* (New Haven & London: Yale University Press), see Chapter 4: Prudent Economy.

24. Elizabeth Kiely & Marie Leane (2008) Money Matters in the Lives of Working Women in Ireland in the 1940s and 1950s, in Francis Devine, Fintan Lane & Niamh Puirseil (Eds) *Essays in Irish Labour History* (Dublin: Irish Academic Press), p. 220. On the skill required for and pride taken in managing domestic resources, see for example Joanne Bourke (1994) *Working-Class Cultures in Britain 1890–1960: gender, class and ethnicity* (London: Routledge), pp. 62–67; Ellen Ross (1993) *Love and Toil: motherhood in outcast London, 1870–1918* (Oxford: Oxford University Press), pp. 40–55; Deirdre Beddoe (1989) *Back to Home and Duty: women between the wars, 1918–1939* (London: Pandora), pp. 99–101.

25. Dorothy Manchée (1946) *Whatever Does the Almoner Do?* (London: Bailliere, Tindall & Cox), preface.

26. Ibid. pp. 8–9.

27. S. Clayton Fyers, ‘The Almoner as seen by the Administrator’, *The Almoner* (June 1948), MRC, MSS.378/IMSW/A/17/9/1, p. 51.

28. H. E. R., ‘Pitfalls of Progress’, *The Almoner* (August 1948), MRC, MSS.378/IMSW/A/19/9/1, p. 89.

29. Miss H. M. Johns, Special Tyneside Scheme, ‘Letter to Miss Steel, Secretary of the Institute of Almoners’, 16 May 1946, MRC, MSS.378/IMSW/A16/2/6.

30. Aneurin Bevan, ‘The Minister of Health Addresses the Annual General Meeting’, *The Almoner* (April 1948), p. 5, MRC, MSS.378/IMSW/A/17/9/1.

31. Wilson Jameson, ‘Message from the Chief Medical Officer of Health’, *The Almoner* (April 1948).

32. I.F. Beck (1946) *The Almoner: A Brief Account of Medical Social Service in Great Britain* (London: Victor Gollancz), p. 60.

33. Ibid. p. 4.

34. Hospital Almoners’ Association [hereafter HAA], ‘Medico-Social Service in a National Health Scheme’, April 1945, MRC, MSS.378/IMSW/A16/1/5, p. 1.

35. Ibid. p. 4.

36. Ibid. p. 3.

37. HAA, ‘The White Paper on a National Health Service: response to consultation’, April 1945, MRC, MSS.378/IMSW/A16/1/4.

38. HAA, ‘The White Paper on a National Health Service: responses to questionnaire 1’, 1944, MRC, MSS.378/IMSW/A16/1/2.

39. HAA, ‘Consultation’.

40. Ibid.

41. HAA, ‘The White Paper on a National Health Service: responses to questionnaire 2’, 1944, MRC, MSS.378/IMSW/A16/1/3.

42. J. Baker, Almoner at the Sunderland Royal Infirmary, ‘Letter to Miss Steel, Secretary of the Institute of Almoners’, 11 May 1946, MRC, MSS.378/IMSW/A16/2/4.

43. Anon. (1935) *The Hospital Almoner: A Brief Study of Hospital Social Service in Great Britain* (London: Hospital Almoners Association), p. 47.

44. Beck, *The Almoner*, p. 5.

45. Ibid. pp. 8–9; *Almoner: Brief Study*, p. 47.

46.
*Almoner: Brief Study*, p. 41.

47. Ibid., p. 61; Steven Cherry (1996) Accountability, Entitlement, and Control Issues and Voluntary Hospital Funding c1860-1939, *Social History of Medicine*, 9(2), p. 230.

48. Rayner Garner, from the UK Midwifery Archives on *Midwifery Matters*, the website of the Association of Radical Midwives, http://www.midwifery.org.uk/?page_id=655 (accessed 25 April 2016).

49. Bella Aronovitch (1974) *Give it Time: an experience of hospital, 1928–1932* (London: Andre Deutsch) cited in Deborah Brunton (Ed.) (2004) *Health, Disease and Society in Europe 1800–1930: a source book* (Manchester: Manchester University Press), p. 285.

50. Lucinda McCray Beier (2008) *For Their Own Good: the transformation of English working-class health culture, 1880–1970* (Columbus: Ohio State University Press), p. 133.

51. Jim Cobley, ‘Happy Birthday NHS’, *TSSA (Transport and Salaried Staffs’ Association) Journal* (August/September 2008), http://www.tssa.org.uk/article-269.php3?id_article=4349 (accessed 9 September 2010).

52. Bristol General Hospital, *Report for 1921*, p. 17; Bristol Royal Infirmary, *Reports for 1921–1938*.

53. Manchée, *Whatever Does the Almoner Do?*, p. 9.

54. D. A. Dow, M. M. Leitch & A. F. MacLean (1982) *From Almoner to Social Worker: social work at Glasgow Royal Infirmary, 1932–1982* (Glasgow: Glasgow Royal Infirmary), p. 10.

55. Beck, *The Almoner*, p. 61.

56. See, for example, Bristol Royal Infirmary, *Report for 1921*, p. 20.

57. MRC, MSS.378/IMSW/A/15/13/4a: Institute of Almoners, Report on Replies to the Questionnaire, ‘The Functions of an Almoner’, 1947.

58. A 1947 questionnaire from the Institute of Almoners, responded to by 233 hospitals, found 0.5% of almoner's departments collecting medical fees from private patients and none being assisted in doing so. When it came to collecting maintenance fees from private patients, almoners were much more likely to be involved. Fourteen percent of departments collected those fees, with 9.5% saying this was done by the almoner, 2.5% a clerk and 2% an administrative assistant. MRC, MSS.378/IMSW/A/15/13/4a: Institute of Almoners, Functions Committee.

59. Martha Vicinus (1985) *Independent Women: work and community for single women, 1850–1920* (London: Virago), p. 86.

60. Vanessa Heggie (2015) Women Doctors and Lady Nurses: class, education, and the professional Victorian woman, *Bulletin of the History of Medicine*, 89(2), pp. 271–272.

61. See Thomson, ‘Between Separate Spheres’; Mary Ann Elston (2001) Run by Women, (Mainly) for Women: medical womens hospitals in Britain, 1866–1948, in Anne Hardy & Lawrence Conrad (Eds) *Women and Modern Medicine* (Amsterdam: Rodopi), pp. 73–108; Maxine Rhodes (2004) Women in Medicine: doctors and nurses, 1850–1920, in Deborah Brunton (Ed.) *Medicine Transformed: health, disease and society in Europe 1800–1930* (Manchester: Manchester University Press), pp. 151–179.

62. Heggie, ‘Women Doctors’, 272.

63. R. Saunderson (1931) An Outside Impression of Hospitals and Their Almoners, in *The Year Book of the Hospital Almoners Association*, vol. 5, p. 25.

64. Beck, *Almoner*, pp. 49–50; *Hospital Almoner: A Brief Study*, p. 34.

65. Alan Moncrieff, ‘Preface’ in Beck, *Almoner*, p. 3.

66. Almoner's Report in the Addenbrooke's Hospital, Cambridge, *Report for 1934–1935*, p. 18.

67. Elizabeth Gloyne (1981) Social Workers Speak Out, 8, MRC, http://www2.warwick.ac.uk/services/library/mrc/explorefurther/speakingarchives/socialwork/929.publ_no_9_gloyne.pdf.

68. Enid Warren (1981) Social Workers Speak Out, 13, MRC, http://www2.warwick.ac.uk/services/library/mrc/explorefurther/speakingarchives/socialwork/929.publ_no_21_warren.pdf.

69. Jean Snelling (1980) Social Workers Speak Out, 21, MRC, http://www2.warwick.ac.uk/services/library/mrc/explorefurther/speakingarchives/socialwork/929.publ_no_18_j_snelling.pdf.

70. The National Archive [hereafter TNA], MH 123/466, Letter from the Ministry of Health, ‘National Health Service: Proposed Charges for Certain Hospital Appliances and Repairs of Surgical Footwear for Outpatients’, 1 April 1952.

71. Ibid.

72. TNA, MH 123/466, Windsor Group No. 4 Hospital Management Committee, ‘Proposed Charges for Certain Hospital Appliances and Repairs of Surgical Footwear for Outpatients’.

73. MRC, MSS.378/IMSW/A/16/4/1: Institute of Almoners, ‘Important Statement’, 21 February 1952. Original emphasis.

74. TNA, MH 123/466, Letter from the Secretary of the Institute of Almoners to the Ministry of Health, ‘Proposed Charges for Hospital Appliances, etc.’, May 1952.

75. TNA, MH 123/466, Letter from the Ministry of Health, ‘National Health Service: Proposed Charges for Certain Hospital Appliances and Repairs of Surgical Footwear for Outpatients’, 1 April 1952.

76. TNA, MH 123/466, Institute of Almoners memorandum, ‘Ministry of Health circulars dealing with charges for prescriptions and certain appliances’, 29 May 1952.

77. TNA, MH 123/466, Note from Mr Nicol to Sir George Godber, 2 June 1952.

78. MRC, MSS.378/IMSW/A/16/4/2: Institute of Almoners, ‘Report of the Emergency Professional Meeting Held on 19th April 1952’.

79. MRC, MSS.378/IMSW/A/16/4/8: Telegram from Ministry of Health to the Institute of Almoners, ‘proposed Charges for Hospital Appliances, etc.’, 21 May 1952.

80. TNA, MH 89/98, ‘Report on Work of the Almoner's Department of the United Newcastle upon Tyne Hospitals, 1960’, p. 3.

81. The most important foundational texts in this area were Mark Granovetter (1985) ‘Economic Action and Social Structure: the problem of embeddedness’, *American Journal of Sociology*, 91(3), pp. 481–510 and Viviana A. Zelizer (1994) *The Social Meaning of Money: pin money, paychecks, poor relief, and other currencies* (New York: Princeton University Press). For an introductory overview, see Geoffrey Ingham (2004) *The Nature of Money* (Cambridge: Polity), esp Ch. 3: ‘Money in Sociological Theory.’

82. See Gosling, ‘Open the Other Eye’; *Payment and Philanthropy*.

83. Anne Witz (1990) Patriarchy and Professions: the gendered politics of occupational closure, *Sociology*, 24(4), pp. 686–687.

84. Ibid. pp. 680–682.

85. Ibid.

